# Genotyping of UGT1A1*80 as an Alternative to UGT1A1*28 Genotyping in Spain

**DOI:** 10.3390/pharmaceutics14102082

**Published:** 2022-09-29

**Authors:** Adrián Bravo-Gómez, Sara Salvador-Martín, Paula Zapata-Cobo, María Sanjurjo-Sáez, Luis Andrés López-Fernández

**Affiliations:** 1 Servicio de Bioquímica, Hospital General Universitario Gregorio Marañón, 28007 Madrid, Spain; 2 Servicio de Farmacia, Instituto de Investigación Sanitaria Gregorio Marañón, Hospital General Universitario Gregorio Marañón, 28007 Madrid, Spain

**Keywords:** genotyping, pharmacogenetics, irinotecan, personalized medicine, cancer

## Abstract

Background: The variant rs34983651 (UGT1A1*28) and its genotyping are used to prevent irinotecan-induced toxicity. Several variants are in close linkage disequilibrium. Our objective was to evaluate the potential correlation of genotyping UGT1A1*80 instead of UGT1A1*28 in different populations. Methods: We studied SNPs in linkage disequilibrium with UGT1A1*28 in several populations and selected rs887829 to develop an inexpensive and rapid genotyping method and compare it with the one we currently use for UGT1A1*28 genotyping. Samples from cancer patients (n = 701) already tested using PCR and electrophoresis prior to treatment with irinotecan for rs34983651 (UGT1A1*28) in a Spanish hospital were genotyped for rs887829 (UGT1A1*80) using real-time PCR with a TaqMan probe. Results: We observed a complete match for both genotypes, except in one sample. This method was 100% efficient in correctly genotyping *28/*28 patients, 99.68% efficient for *1/*28, and 100% efficient for *1/*1. Linkage disequilibrium between populations showed the Iberian population to be the most suitable for the clinical use of UGT1A1*80. This method is less expensive and the time to decision is shorter. Conclusion: Genotyping of rs887829 using the proposed method may be used to substitute genotyping of rs34983651 as a pharmacogenetics test in cancer patients prior to starting irinotecan-based treatments, mainly in the Iberian population. In addition, it is less expensive than other conventional methods and easy to implement, with a shorter time to decision than UGT1A1*28.

## 1. Introduction

The uridine diphosphate glucuronosyltransferases (UGT1) are a family of enzymes involved in the glucuronidation of multiple substrates. UGT1A1 participates in the glucuronidation of SN38, a metabolite with antiproliferative activity resulting from the metabolization of irinotecan. Severe diarrhea and neutropenia are common adverse events of irinotecan [1]. Several genetic variants in UGT1A1 have been associated with toxicity induced by irinotecan [2] and by other cancer drugs [3].

The most studied variant is a tandem repeat of TA in the promoter region of *UGT1A1* that regulates the expression of *UGT1A1* mRNA [4]. Thus, six TA repeats is the most frequent condition and is considered normal expression. However, seven TA repeats (UGT1A1*28) and eight TA repeats (UGT1A1*37) are associated with reduced expression of UGT1A1, lower metabolizing capacity for SN38 and other drugs, and increased frequency of drug-induced toxicity [5]. UGT1A1*28 is the most common cause of Gilbert syndrome and is usually diagnosed by genotyping.

The variant rs887829 c.-364C>T (UGT1A1*80) has no known effect on the expression of UGT1A1. However, it is in high linkage disequilibrium (LD) with UGT1A1*28. Based on this association, the CPIC guidelines for atazanavir and *UGT1A1* state that if only UGT1A1*80 is interrogated and the patient is heterozygous or homozygous for *80, an intermediate or poor metabolizer phenotype, respectively, may be inferred [6]. This recommendation is based exclusively on the global high LD between these two SNPs. However, various questions need to be addressed before this test can be implemented in clinical practice. Firstly, what is the error rate when UGT1A1 metabolic status is inferred by UGT1A1*80 instead of *28 genotyping? Is it clinically relevant? What are the positive and negative predictive values? Secondly, there is no recommendation on UGT1A1*80 genotyping to avoid irinotecan-induced toxicity in cancer patients. Currently, only two guidelines—the Dutch Pharmacogenetics Working Group (DPWG) [7] and the French Network of Pharmacogenetics (RNPGx) [8]—have been developed for *UGT1A1* genotyping and treatment with irinotecan. Neither of these guidelines addresses the possibility of testing UGT1A1*80 as an alternative to *28. Thirdly, there are no studies about differences in LD between different populations or the clinical consequences of these differences. Fourthly, methods for genotyping of UGT1A1*28 and *80 in clinical practice have not been compared.

This study aims to evaluate the potential correlation of genotyping UGT1A1*80 instead of UGT1A1*28 in different populations and to develop a cheap and quick method for UGT1A1*80 genotyping.

Several methods have been developed to genotype UGT1A1*28. In our hospital, we genotype *UGT1A1* using PCR with a fluorescently-labeled oligonucleotide and high-resolution electrophoresis [9]. We compared methods for and the results of genotyping UGT1A1*80 in a population of cancer patients previously tested for UGT1A1*28 prior to selection of treatment in daily clinical practice. Cost and time to decision are compared between the different methodologies.

## 2. Materials and Methods

*Samples*. Blood samples from 701 patients with different types of cancer were genotyped for UGT1A1*28 in the Laboratory of Pharmacogenetics using a standard test before deciding on chemotherapy. These samples were used to genotype UGT1A1*80 after approval by the local ethics committee.

UGT1A1*28 (rs34983651) Genotyping. DNA was isolated using the Maxwell RSC Blood DNA kit (Promega, Fitchburg, WI, USA) and quantified in a Quawell-5000 spectrophotometer (Quawell, Palo Alto, CA, USA). This SNP was genotyped using amplified fragment length polymorphism with the following primers: UGT28-F, 6FAM-TCACGTGACACAGTCAAACATT; and UGT28R, CAGCATGGGACACCACTG. PCR conditions and reactions were as described in Cortejoso et al. [9]. PCR products were purified using the High Pure PCR Product Purification kit (Roche Life Science, Basel, Switzerland). The amplicon was loaded into an ABI 3130XL Genetic Analyzer and analyzed using PeakScanner (Applied Biosystems, Carlsbad, CA, USA). Sanger sequenced controls for *UGT1A1* *1/*1, *1/*28 and *28/*28 were used in all tests.

UGT1A1*80 (rs887829) Genotyping. A 3-µL aliquot of blood was lysed in 10 µL NaOH 0.2N at 75 ºC and then mixed with 90 µL of neutralizing solution (40 mM Tris-HCl, 0.55 mM EDTA pH 8.0). The sample was ready for genotyping after centrifugation at 14,000 r.p.m. for 1 min. The region containing the SNP rs887829 was then genotyped using real-time PCR in QuantStudio 3 (Applied Biosystems) with a TaqMan probe (C__2669357_10) and TaqPath ProAmp Master mix (Applied Biosystems) following the manufacturer’s instructions, except that the final reaction volume was 5 µL instead of 10 µL. Allelic discrimination plots were used to assign a genotype.

*Calculation of linkage disequilibrium*. The LDlink tool from the National Cancer Institute was used to analyze LD [10]. Genetic variants in LD with rs34983651 were calculated using the LDproxy tool. LD for rs887829 and rs34983651 was calculated in different populations using the LDmatrix tool in a 1000-genome dataset (Caucasian European [CEU] population). In addition, the LD was calculated for these 2 SNPs in the study population (n = 701).

The positive predictive value (PPV), negative predictive value (NPV), specificity, and sensitivity were calculated online (https://mathcracker.com, accessed on 1 September 2022).

*Genotyping costs.* The total cost of *UGT1A1* genotyping was calculated based on the cost of reagents, equipment, and personnel involved. The amortization period was estimated at 10 years. PCR costs were estimated based on 3 samples with different genotypes as controls and a negative control without DNA or a lysed sample. For equipment, cost was calculated based on a 10-year amortization period and the number of pharmacogenetic tests performed in our laboratory in 1 year (2021).

## 3. Results

### 3.1. Linkage Disequilibrium Analyses

The LDproxy Tool revealed 856 entries to be associated with the functional variant rs34983651. The most related SNP based on LD parameters (D’ = 0.9924; R^2^ = 0.8663) was rs887829, an intronic variant. Allele C rs887829 correlated with 6 TA repeats in rs24983651 and allele T correlated with 7 TA repeats (Table 1).

The LD between rs3483651 and rs887829 was analyzed in different populations using 1000 genomes and the GrCh37 version of the genome. Differences in correlation (R^2^) were observed between the populations. The cohort with the highest correlation between these two SNPs was the Iberian population (R^2^ = 0.9772, D’ = 1.0), whereas the lowest correlation was observed for the African cohort (R^2^ = 0.7646, D’ = 1.0) (Table 2).

Our results revealed LD between rs34983651 and rs887829. The rs34983651(-) allele was correlated with the rs887829(C) allele, and the rs34983651(AT) allele was correlated with the rs887829(T) allele.

### 3.2. Real Predictive Values for rs887829 Genotyping

Genotyping of both polymorphisms showed an extremely high correlation across the 701 samples. For UGT1A1*80, 312 samples were inferred to A(TA)_6_TAA in homozygosis, 308 to A(TA)_6_TAA/A(TA)_7_TAA in heterozygosis, and 81 to A(TA)_7_TAA in homozygosis. For UGT1A1*28, 313 samples were genotyped as homozygous A(TA)_6_TAA, 307 as heterozygous A(TA)_6_TAA/A(TA)_7_TAA, and 81 as homozygous A(TA)_7_TAA. Discrepancies between the methods were observed for only one sample, which was heterozygous for the UGT1A1*80 genotype and homozygous A(TA)_6_TAA for UGT1A1*28.

This method was 100% efficient in correctly genotyping *28/*28 patients, 99.68% efficient for *1/*28 patients, and 100% efficient for *1/*1 patients. In addition, this technique showed 100% efficacy for detecting the A(TA)_6_TAA allele and 99.79% efficacy for detecting the A(TA)_7_TAA allele.

The predictive values for rs887829 genotyping and correlation with UGT1A1*28 were calculated for the study cohort. The positive predictive value (PPV), negative predictive value (NPV), sensitivity, and specificity were >99.5% in all cases (Table 3).

### 3.3. Comparison of Genotyping Cost and Time to Result

The costs of UGT1A1*28 and UGT1A1*80 genotyping were calculated and compared (Table 4). Fragment analysis of UGT1A1*28 had a total cost per sample of EUR 17.32 when 10 samples were genotyped simultaneously. The cost of reagents was EUR 13.19/sample, amortization of equipment based on 1000 samples genotyped per year was EUR 0.6/sample, and cost of time needed to proceed with this analysis was EUR 3.53/sample. This cost increased if fewer than 10 samples were genotyped simultaneously (EUR 20.8 and EUR 41.76/sample processing five and one sample, respectively).

TaqMan genotyping for UGT1A1*80 cost EUR 4.21/sample when 10 samples were genotyped per run. The cost of reagents was EUR 1.16/sample, the amortization of equipment based on 1000 samples genotyped per year was EUR 2/sample, and the cost of time to procced with the analysis was EUR 1.05/sample. This cost increased if fewer than 10 samples were genotyped simultaneously (EUR 2.11 and EUR 16.71/sample processing five or one sample, respectively).

Remarkably, the cost of genotyping was 2.50–4.11-fold higher for fragment analysis of UGT1A1*28 than for real-time genotyping of UGT1A1*80. This difference is mostly due to the cost of reagents, which is 4.84–11.37-fold higher for UGT1A1*28 genotyping than for *80 genotyping.

*Genotyping cost of commercial kits:* We compared the cost of our method with an available commercial kit for in vitro diagnostics (*UGT1A1* Genotyping kit for Real-Time PCR [EntroGen, Woodland Hills, CA, USA]). This comparison showed an 11-fold higher cost when 10 samples were genotyped per run.

The time spent on each of the stages of both methods is shown in Table 5. Analysis of UGT1A1*28 by PCR and electrophoresis takes 1 day 1 h and 15 min per sample, increasing to 1 day and almost 2 h for 10 samples. However, the time required for analysis of UGT1A1*80 using TaqMan probes is considerably shorter. The time between receipt of the sample and generation of the result is 45 min per sample, rising to 1 h and 10 min for 10 samples.

## 4. Discussion

UGT1A1*28 is clearly associated with severe neutropenia and diarrhea in patients treated with irinotecan, mainly in homozygous patients [11]. However, whereas this is not the only genetic variant in *UGT1A1* that makes it possible to predict irinotecan-induced toxicity, it is the most widely genotyped polymorphism for the prevention of irinotecan-induced severe adverse reactions in clinical practice.

Several authors describe the use of TaqMan probes to genotype UGT1A1*28 [12,13]. In our experience, this method is not as robust as it should be in clinical practice, since it does not easily discriminate a 2 insdel. In fact, a TaqMan probe designed by the owner of this technology did not pass their internal quality control for correct genotyping and was not supplied. Therefore, we sought an alternative in other polymorphisms that were highly linked to UGT1A1*28. In this sense, the SNP rs887829 (UGT1A1*80) is mentioned in the most recent update of the guideline of the Clinical Pharmacogenetics Implementation Consortium (CPIC) for atazanavir and *UGT1A1* [6].

We demonstrated that rs887829 (UGT1A1*80) is in high LD with rs34983651 (UGT1A1*28) and that the Iberian population is the most suitable for clinical implementation of UGT1A1*28-inferred genotype by UGT1A1*80 genotyping. Using the method described here, analysis of detection of UGT1A1*80 in 701 patients previously genotyped for UGT1A1*28 showed a unique non-match: an A(TA)_6_TAA/A(TA)_7_TAA sample was considered A(TA)_6_TAA/A(TA)_6_TAA. The predictive value of UGT1A1*28 is low when the patient is not homozygous for UGT1A1*28 [14,15]. In addition, drug labels and most of the guidelines from groups of experts highly recommend reducing irinotecan doses only in *28/*28 patients, but not in heterozygous patients [7,16]. From this point of view, the mismatch in our cohort did not have any clinical consequence, since all patients who were homozygous for UGT1A1*28 were correctly genotyped.

Several methods have been developed in recent years, and up to eight different methods for rs3064744 genotyping were recently compared [17]. Mismatches were observed between the different methodologies. Thus, the DMET Plus array misclassified 9 (TA)_6_/(TA)_7_ as (TA)_6_/(TA)_6_ in 163 samples, PharmacoScan generated an incorrect result in one sample, and pyrosequencing was not able to conclude a genotype in seven samples. Since we ran 701 genotypes and only one mismatch was found between UGT1A1*28 and UGT1A1*80, the method described here, as well as the UGT1A1*28 genotype inferred by the UGT1A1*80 genotype, is more effective than other methods that specifically genotype UGT1A1*28.

In our laboratory, we routinely use PCR of a region containing the polymorphism with two primers, one of which is labeled with a fluorescent molecule, followed by high-resolution electrophoresis [9]. Although this method of genotyping UGT1A1*28 is effective, it requires a sequencer, which is not commonly available in many laboratories or in small hospitals. However, the method we describe requires real-time PCR, which is more readily available in small laboratories and hospitals.

Our method constitutes a quick, inexpensive, and efficient approach for detecting individuals at risk of severe adverse reactions to irinotecan. It could help to increase cost-effectiveness by reducing the cost of genotyping since UGT1A*80 can be genotyped for only EUR 4.21. The cost-effectiveness of *UGT1A1* genotype-guided dosing of irinotecan was recently demonstrated based on a genotyping cost of EUR 83/patient [18]. However, Hulsof et al. genotyped two variants (UGT1A1*28 and UGT1A1*93), and our method was only one. Consequently, the increased cost of genotyping two variants with our method would be as much as EUR 8.42, which is only 10% of their genotyping cost.

The analysis of LD for rs34983651 and rs887829 between different populations showed that for all the population studies, this link was strongest in the Iberian population. Consequently, the implementation of the rs34983651-inferred genotype by rs887829 genotyping is more suitable. In contrast, the weakest link was observed for Asian and African populations, thus indicating that rs887829 genotyping should not be recommended to avoid irinotecan-induced toxicity. The evaluation of differences in LD is a useful way to establish differences between populations [19]. One limitation of our study is that our results were based on the 1000-genome dataset. However, differences could be found in larger datasets. Unfortunately, datasets such as gnomAD do not allow the analysis of LD, and if they did, differences might be found when an in-depth pharmacogenetic analysis is performed [20].

Another limitation is that in our cohort, we did not have samples with five or eight TA repeats. We do not have data about the correlation between rs887829 and these very rare variants. In our experience, after more than 3000 samples were genotyped for UGT1A1*28, only one sample with five TA repeats was found. However, we consider the clinical relevance of this limitation to be extremely low.

## 5. Conclusions

The genotyping of rs887829 using the proposed method shows a very high correlation with rs34983651, especially in the Iberian population. Since the last variant is genotyped to personalize irinotecan-based therapy, our find suggests that the genotyping of rs887829 could be an alternative to be used in clinical practice. It is also less expensive than other conventional methods and easy to implement, and the time to decision is shorter than for UGT1A1*28.

## Figures and Tables

**Table 1:** Top ten variants in linkage disequilibrium with rs34983651.

*rs* Number	Coordinates	Alleles	MAF ^1^	Distance	D’	R^2^	Correlated Alleles
rs34983651	Chr2:234668879	(-/AT)	0.3253	0	1.0	1.0	-=-,AT=AT
rs887829	Chr2:234668570	(C/T)	354	−309	0.9924	0.8663	-=C,AT=T
rs111741722	Chr2:234665983	(A/G)	0.3536	−2896	0.9896	0.8628	-=A,AT=G
rs4148325	Chr2:234673309	(C/T)	0.3538	4430	0.9876	0.8588	-=C,AT=T
rs35754645	Chr2:234664586	(TC/-)	0.3524	−4293	0.9839	0.8575	-=TC,AT=-
rs6742078	Chr2:234672639	(G/T)	0.3476	3760	0.9652	0.8428	-=G,AT=T
rs4148324	Chr2:234672722	(T/G)	0.3528	3843	0.9659	0.8249	-=T,AT=G
rs3771341	Chr2:234673239	(G/A)	0.3299	4360	0.8928	0.7807	-=G,AT=A
rs10929302	Chr2:234665782	(G/A)	0.3021	−3097	0.9187	0.7579	-=G,AT=A
rs6714634	Chr2:234664765	(T/C)	0.3021	−4114	0.9167	0.7547	-=T,AT=C
rs34352510	Chr2:234650562	(T/C)	0.3303	−18317	0.8781	0.7538	-=T,AT=C

^1^ MAF (minor allele frequency).

**Table 2:** Linkage disequilibrium between rs3483651 and rs887829 in world populations.

R^2^	D’	Chi-sp	*p* Value	Population
0.909558	1.0	180.0925	<0.0001	Utah residents from north and west Europe (CEU) (n = 198)
0.9772	1.0	209.125	<0.0001	Iberian (n = 214)
0.9767	1.0	209.0243	<0.0001	Toscani (n = 214)
0.8932	1.0	176.8601	<0.0001	Finnish (n = 198)
0.9719	1.0	179.8883	<0.0001	Britain (n = 182)
0.9437	0.9855	949.3507	<0.0001	European (CEU, Iberian, Toscani, Finnish, Britain) (n = 1006)
0.7646	1.0	1010.8077	<0.0001	African (n = 1322)
0.9166	0.9936	636.152	<0.0001	Mixed American (Mexican, Puerto Ricans, Colombians, Peruvians) (n = 694)
0.9565	0.9823	964.1246	<0.0001	East China (n = 1008)
0.8404	0.9909	821.8782	<0.0001	South Asia (n = 978)
0.8663	0.0024	4338.4445	<0.0001	All (n = 5008)

**Table 3:** Study of the predictive capacity of UGTA1A*80.

	Homozygous A(TA)_6_TAA	Heterozygous A(TA)_6_TAA/A(TA)_7_TAA	Homozygous A(TA)_7_TAA
**PPV**	99.68%	100%	100%
**NPV**	100%	99.75%	100%
**Sensitivity**	100%	99.67%	100%
**Specificity**	99.74%	100%	100%

**Table 4:** Comparison of genotyping cost by technique used.

Concept	Cost (EUR) 1 Sample per Run	Cost (EUR) 5 Samples per Run	Cost (EUR) 10 Samples per Run
Total cost			
Fragment ^1^	41.8	20.8	17.3
TaqMan ^2^	16.7	5.6	4.2
Commercial ^3^	96.6	52.5	46.8
Ratio Frag/TaqMan	2.5	3.7	4.1
Ratio Commercial/TaqMan	5.8	9.4	11.1
Reagents cost			
Fragment ^1^	20.0	14.1	13.2
TaqMan ^2^	4.1	1.5	1.2
Commercial ^3^	73.4	44.9	41.3
Ratio Frag/TaqMan	4.8	9.8	11.4
Ratio Commercial/TaqMan	17.8	30.1	35.6
Equipment			
Fragment ^1^	0.6	0.6	0.6
TaqMan ^2^	2.0	2.0	2.0
Commercial ^3^	2.0	2.0	2.0
Ratio Frag/TaqMan	0.3	0.3	0.3
Ratio Commercial/TaqMan	1.0	1.0	1.0
Staff			
Fragment ^1^	21.2	5.6	3.5
TaqMan ^2^	10.6	2.1	1.0
Commercial ^3^	21.2	5.6	3.5
Ratio Frag/TaqMan	2.0	2.7	3.4
Ratio Commercial/TaqMan	1.0	1.0	1.0

^1^ Genotyping of UGT1A1*28 by PCR and electrophoresis; ^2^ genotyping of UGT1A1*80 by TaqMan probe using the method described here; ^3^ genotyping using the commercial EntroGen kit.

**Table 5:** Comparison of times expended to complete genotyping.

Task	Time per Sample	Time per 5 Samples	Time per 10 Samples
PCR and electrophoresis ^1^			
DNA extraction	42′	45′	50′
PCR			
Purification	20′	25′	30′
Electrophoresis	1 day	1 day	1 day
Analysis of results	10′	15′	20′
TaqMan probe ^2^			
DNA extraction	5′	7′	10′
Real-Time PCR	35′	40′	45′
Analysis of results	5′	10′	15′

^1^ Genotyping of UGT1A1*28 by PCR and electrophoresis; ^2^ genotyping of UGT1A1*80 by TaqMan probe.

## Data Availability

Not applicable.
